# A Cardiac Tumor and Liver Masses on Point of Care Ultrasound (POCUS): Implications in a Resource-Limited Setting 

**DOI:** 10.24908/pocus.v9i2.17665

**Published:** 2024-11-15

**Authors:** Waseem Sous, George Limwado, Enoch Ndarama, Michaela Sous

**Affiliations:** 1 Division of Hospital Medicine, University of California San Francisco, CA USA; 2 HEAL Initiative, University of California San Francisco, CA USA; 3 Partners in Health/ Abwenzi Pa Za Umoyo Neno MWI; 4 Ministry of Health Neno MWI

**Keywords:** Point-of-Care Ultrasound, Cardiac Tumor, Resource-Limited Setting

## Abstract

Malignant cardiac tumors are quite rare, but portend a poor prognosis 1, 2. Early identification and classification are essential given their aggressive nature, particularly when metastases are present 3, 4. Clinical presentations are varied, and detection relies primarily on echocardiography. Thus, cardiac tumors may go undiagnosed in areas where echocardiography is not routinely available 1, 5. In this case, point of care ultrasound (POCUS) rapidly detected a cardiac mass and liver lesions, prompting referral to a central hospital in Malawi for further evaluation. This case highlighted the potential role of POCUS as a readily available tool in a resource-limited setting, serving as a triage point for more definitive diagnosis and management 6, 7, 8.

## Case Presentation

A 37-year-old man presented to a rural district hospital in Malawi for evaluation of a six-month history of progressively worsening abdominal distention, lower extremity swelling, dyspnea on exertion, and unintentional weight loss. He reported a history of alcohol use disorder for ten years but stopped drinking one year prior to presentation. He had no history of chronic disease, tobacco use, surgeries, or family history of malignancy.

On presentation, the patient was tachycardic, but hemodynamically stable and without acute distress. He appeared cachectic with muscle wasting. He had significant abdominal distention with hepatomegaly extending into the right lower quadrant with tenderness to palpation without guarding, rebound, or rigidity. There was no evidence of splenomegaly on examination. The heart examination revealed a laterally displaced point of maximal impulse and a diastolic murmur at the left lower sternal border. He also had jugular venous distention (JVD) and lower extremity pitting edema. The lung examination was unremarkable. He had no cervical, inguinal, or axillary lymphadenopathy, or skin or mucocutaneous lesions. Laboratory results showed an unremarkable complete blood count, a negative human immunodeficiency virus test, and negative hepatitis B and C tests. The treating physicians were left without a clear diagnosis due to resource limitations. These included lack of access to kidney and liver function testing due to frequent reagent shortages, serum alpha-fetoprotein testing, and computed tomography (CT) scan of the chest, abdomen, and pelvis at the district hospital. In particular, CT imaging would have helped in the assessment of metastatic disease and/or the presence of a tumor thrombus.

**Figure 1:**
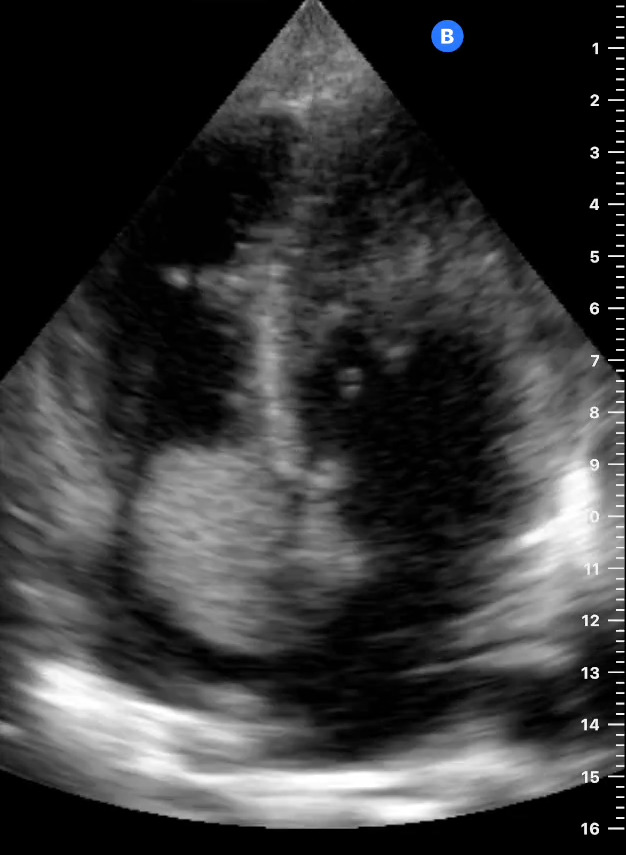
Apical four-chamber view during diastole showing a 4x2 cm right atrial mass (arrow) originating from the interatrial septum with almost complete obstruction of tricuspid valve during atrial systole.

**Figure 2:**
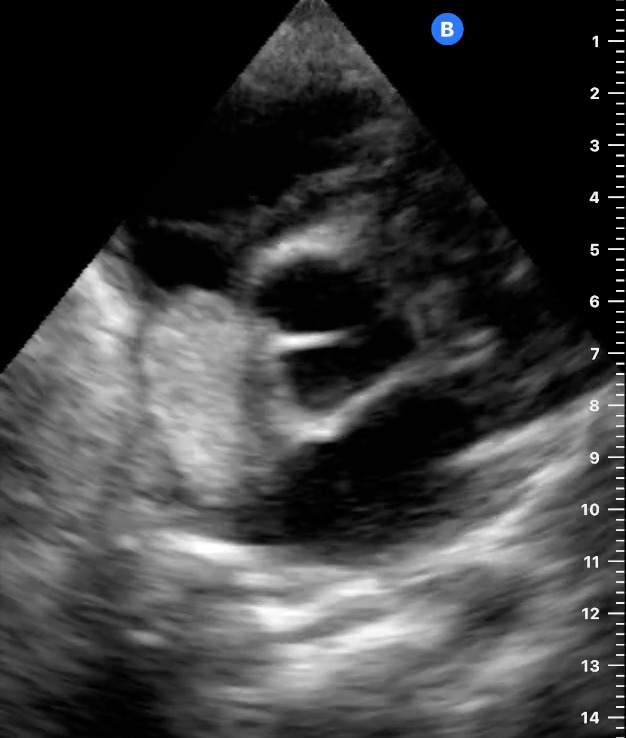
Parasternal short-axis view at the level of the cardiac base showing the right atrial mass (arrow).

**Figure 3:**
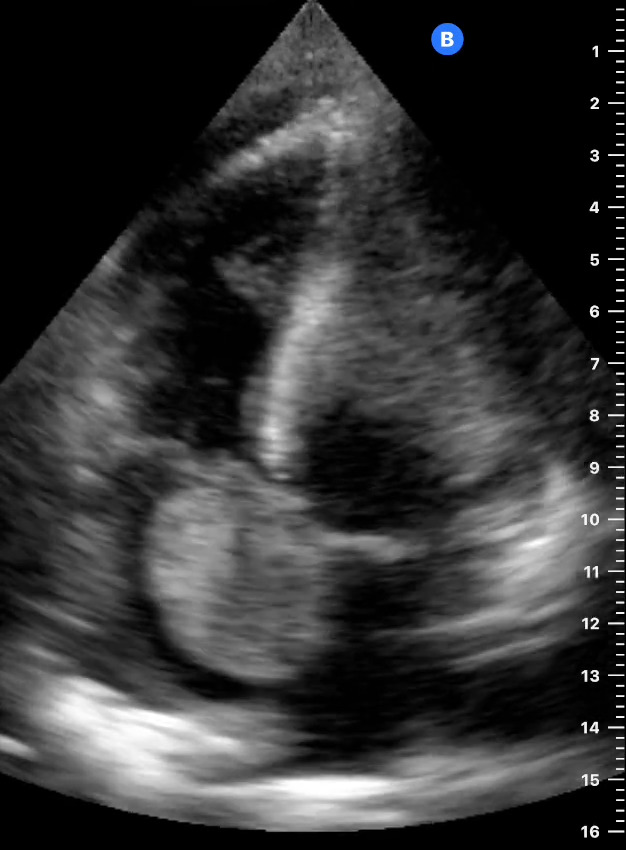
Arrow showing trivial pericardial effusion only seen in systole.

**Figure 4:**
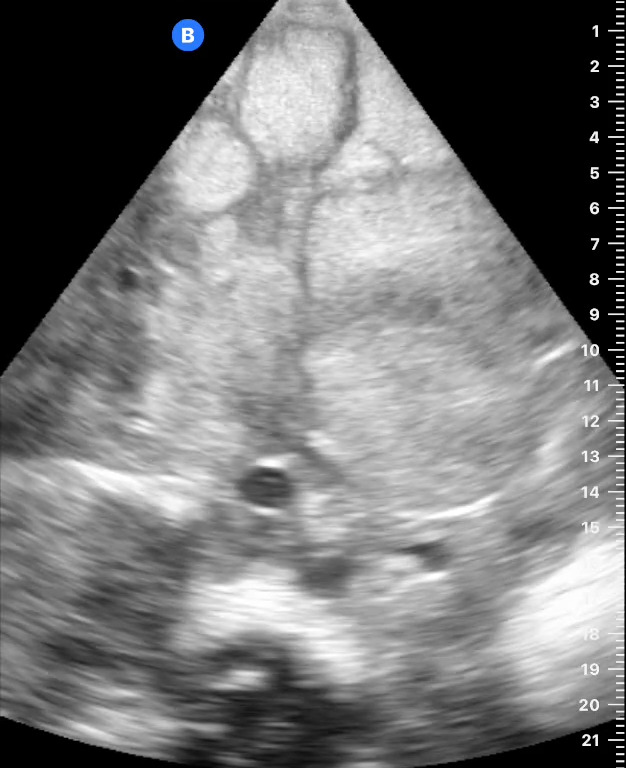
Right upper quadrant view showing multiple hyperechoic liver masses (arrows).

Point of care ultrasound (POCUS) was then pursued. The apical view (Figure 1) and parasternal short-axis view (Figure 2) at the level of the cardiac base showed a 4x2 centimeter right atrial mass originating from the interatrial septum, resulting in tricuspid valve obstruction during atrial systole. Qualitative assessment of the left ventricular function was normal. Although the tricuspid annular plane systolic excursion was not measured, the right ventricular size was less than two-thirds the size of the left ventricle (Figure 1) and there was no evidence of interventricular septal bowing^9^ to suggest right ventricular dysfunction. The apical view (Figure 3) showed evidence of a trivial pericardial effusion only seen in systole 10. Additional assessment of the pericardial effusion with a subcostal cardiac view could not be obtained due to significant hepatomegaly. Abdominal POCUS, as shown in Figure 4, demonstrated multiple hyperechoic hepatic masses of varying sizes without ascites. Given the right atrial mass and liver masses, the patient was referred to the central hospital for further diagnostic evaluation and consideration of treatment for presumed metastatic cancer.

## Discussion

This case emphasized the role of POCUS in early detection and triage in a resource-limited setting 6, 7, 8. While cardiac tumors are rare, they have a high morbidity and mortality, and early identification is essential given their extremely poor prognosis when metastatic disease is present 1, 2, 3, 4. Clinical symptoms vary, and even benign tumors may cause valve obstruction, compression of cardiac chambers, arrhythmias, and embolization^1^ depending on the size, location, and degree of invasion 3.

In this case, history, physical examination, and limited initial laboratory workup failed to identify the cause of the patient’s symptoms. The symptoms of volume overload prompted a stepwise POCUS evaluation to investigate common causes, which in turn identified cardiac and liver masses. The most likely etiology of JVD and lower extremity edema was secondary to the tricuspid valvular obstruction due to the right atrial mass, particularly given the normal qualitative assessment of the left ventricular function and low suspicion for right ventricular dysfunction. Transthoracic and transesophageal echocardiography are typically the initial diagnostic tests for cardiac tumors given the lower sensitivity of POCUS. However, this case demonstrated that POCUS can be a useful diagnostic tool beyond the basic indications (e.g. left ventricular systolic dysfunction and pericardial effusion) and can help make a less common diagnosis when using a symptom-directed POCUS exam 11.

Once a cardiac mass is identified, the diagnostic approach involves differentiating cardiac tumors from thrombi or vegetations through echocardiography 1. Common intracardiac tumors include myxomas, papillary fibroelastomas, and angiosarcomas 1. A myxoma is most frequently detected in the left atrium, while a papillary fibroelastoma is most seen on cardiac valves 1. An angiosarcoma, the most frequent primary malignant tumor, usually originates from the right atrial walls including the interatrial septum 1. In our case, the right atrial mass appeared to originate from the interatrial septum. However, we were unable to obtain additional views to assess if the cardiac mass was extending from the inferior vena cava into the right atrium, which can be seen with a thrombus or a tumor thrombus 1. Nevertheless, identification of liver masses made metastatic cardiac angiosarcoma the most likely diagnosis 3, 4, 12, 13. Additional differential diagnoses for an atrial mass and liver lesions include a hepatic mass with a tumor thrombus in the right atrium,^14^ direct extension of hepatocellular carcinoma into the right atrium,^15^ hepatocellular carcinoma with metastasis to the heart,^16^, ^17^ and synchronous hepatocellular carcinoma and cardiac myxoma 18. Cases of hepatocellular carcinoma would be epidemiologically relevant in Malawi given the high prevalence of hepatitis B 19. However, hepatocellular carcinoma was less likely for this patient given the negative hepatitis B and C testing.

Furthermore, we acknowledge that POCUS should not be utilized to rule out cardiac tumors. Adequate assessment of cardiac tumors is dependent on machine quality and provider skills. In this case, POCUS provided useful information that aided in early detection, triage, and subsequent transfer to the nearest central hospital for further diagnostic evaluation and management.

## Conclusion

This case demonstrated the ability of POCUS to diagnose intracardiac tumors and associated findings to formulate an appropriate management plan in a resource-limited setting 6, 7, 8. In general, POCUS is less sensitive for tumor identification in both the heart and liver and may lack the ability to differentiate the type of intracardiac tumor due to device and operator limitations. Although POCUS may not be able to rule out intracardiac pathology, POCUS can speed detection of some intracardiac tumors and can facilitate timely referral for more definitive diagnosis and management.

## Patient Consent

The patient gave informed consent for this case file.

## Conflicts of Interest

Authors declare that they have no conflict of interest. No financial or material support was provided to the authors.

## Supplementary Material

Video S1

Video S2

Video S3
